# Activating Invasion and Metastasis in Small Cell Lung Cancer: Role of the Tumour Immune Microenvironment and Mechanisms of Vasculogenesis, Epithelial‐Mesenchymal Transition, Cell Migration, and Organ Tropism

**DOI:** 10.1002/cnr2.70018

**Published:** 2024-10-07

**Authors:** Carl He

**Affiliations:** ^1^ Department of Oncology, Eastern Health University of Melbourne Melbourne Australia

**Keywords:** epithelial‐mesenchymal transition, lung cancer, metastasis, small cell lung cancer

## Abstract

**Background:**

Small cell lung cancer (SCLC) harbours the most aggressive phenotype of all lung cancers to correlate with its bleak prognosis. The aggression of SCLC is partially attributable to its strong metastatic tendencies. The biological processes facilitating the metastasis in SCLC are still poorly understood and garnering a deeper understanding of these processes may enable the exploration of additional targets against this cancer hallmark in the treatment of SCLC.

**Recent Findings:**

This narrative review will discuss the proposed molecular mechanisms by which the cancer hallmark of activating invasion and metastasis is featured in SCLC through important steps of the metastatic pathway, and address the various molecular targets that may be considered for therapeutic intervention. The tumour immune microenvironment plays an important role in facilitating immunotherapy resistance, whilst the poor infiltration of natural killer cells in particular fosters a pro‐metastatic environment in SCLC. SCLC vasculogenesis is achieved through VEGF expression and vascular mimicry, and epithelial‐mesenchymal transition is facilitated by the expression of the transcriptional repressors of E‐cadherin, the suppression of the Notch signalling pathway and tumour heterogeneity. Nuclear factor I/B, selectin and B1 integrin hold important roles in SCLC migration, whilst various molecular markers are expressed by SCLC to assist organ‐specific homing during metastasis. The review will also discuss a recent article observing miR‐1 mRNA upregulation as a potential therapeutic option in targeting the metastatic activity of SCLC.

**Conclusion:**

Treatment of SCLC remains a clinical challenge due to its recalcitrant and aggressive nature. Amongst the many hallmarks used by SCLC to enable its aggressive behaviour, that of its ability to invade surrounding tissue and metastasise is particularly notable and understanding the molecular mechanisms in SCLC metastasis can identify therapeutic targets to attenuate SCLC aggression and improve mortality.

## 1 Background

Lung cancer is the foremost contributor to cancer mortality worldwide [1]. Lung cancer is broadly classified as small cell lung cancer (SCLC) and non‐small cell lung cancer (NSCLC), [2] with SCLC accounting for 15% of all lung cancers [3].

SCLC, a neuroendocrine tumour of the lung, [4] is the most aggressive lung cancer subtype, [5] characterised by rapid proliferation and metastasis and frequent chemoresistance [6]. Recently, SCLC was subclassified into the subtypes of SCLC‐A, N, P and Y according to their high expression of the transcription factors Achaete‐scute homologue 1 (ASCL1), neurogenic differentiation 1 (NEUROD1), POU class 2 homeobox 3 (POU2F3) and yes‐associated protein 1 (YAP1), respectively [7]. ASCL1 and NEUROD1 are key regulators of neuroendocrine differentiation, conferring a greater neuroendocrine phenotype to SCLC‐A and SCLC‐N, respectively, which together comprise approximately 80% of SCLCs [8]. It is known that the molecular subtypes have some differences in their gene expression profiles, [9] however, there is limited research into the mechanisms of tumourigenesis and the potential therapeutic targets of SCLC as stratified by their molecular subtypes. Due to the differential gene expressions of the subtypes, molecular characterisation of SCLC in future research will be necessary, to identify more effective and specific treatments.

SCLC carries a poor prognosis, with a 5‐year overall survival (OS) rate under 7% [5]. There has been minimal therapeutic advancement in SCLC for several years, and etoposide combined with platinum‐based chemotherapy remains as standard treatment in all patients [10]. Unfortunately, chemoresistance to this regimen often occurs within 1 year of treatment, resulting in disease recurrence [10].

## 2 Therapeutic Resistance Mechanisms and the Tumour Immune Microenvironment

Multiple means of chemoresistance may be adopted by SCLC. Proteomic studies have identified an overexpression of DNA repair proteins in SCLC lines relative to NSCLC, including PARP1, X53BP1, DNA‐PKcs and ATM, which likely reduce the efficacy of DNA‐damaging agents such as etoposide and platinum‐based chemotherapy [11, 12]. Patient‐derived xenograft (PDX) modelling of SCLC cisplatin and etoposide resistance by Gardner et al. identified a pattern of SLFN11 gene suppression and TWIST1 gene upregulation in resistant cell lines [13]. SLFN11 is a DNA/RNA helicase that irreversibly blocks replication fork progression, [14] while TWIST1 is implicated in stemness, epithelial‐mesenchymal transition (EMT) and metastasis [12]. The Wnt/B‐catenin signalling pathway is implicated in the chemoresistance of multiple tumour types, including 1%–3% of SCLCs. Activation of the Wnt/B‐catenin pathway induces the production of drug‐resistant proteins including multiple ATP‐binding cassette subfamily members, while also activating downstream anti‐apoptotic genes including CyclinD1, Bcl‐2 and survivin [15]. Accordingly, the Interferon‐induced transmembrane protein 1 (IFITM1) molecule was recently shown to be overexpressed in cisplatin‐resistant SCLC cells, with IFITM1 functioning as an activator of the Wnt/B‐catenin pathway [15]. An analysis by Drapkin et al. on SCLC PDX models treated with platinum chemotherapy and etoposide also identified an association between the Myc gene signature and resistance [16]. While Myc has a known role in tumour proliferation and metastasis, further research is needed to elucidate the mechanisms underlying its role in SCLC chemoresistance.

SCLCs are also challenging targets for immunotherapy, due to their complex tumour immune microenvironment (TIME). The anti‐PD‐L1 immune checkpoint inhibitors atezolizumab and durvalumab were approved by the U.S. Food and Drug Administration (FDA) as additional agents to the etoposide‐platinum chemotherapy backbone in extensive‐stage SCLC in 2019 and 2020, following the IMpower133 (atezolizumab) and CASPIAN (durvalumab) trials, respectively. These trials, however, were only able to demonstrate a small increase in the median OS compared to the standard etoposide‐platinum chemotherapy control with increases of 2.0 months (IMpower133) and 2.7 months (CASPIAN) [17, 18]. The challenge of targeting SCLC with immunotherapy and the SCLC propensity for immune evasion may partially be attributed to the TIME of SCLCs, particularly the subtypes with the neuroendocrine phenotype, which demonstrate poor immunogenicity [19]. SCLC has diminished antigen‐presenting mechanisms, with 71% expressing little to no MHC I [20] and no SCLCs expressing MHC II, [21] resulting in reduced cytotoxic T lymphocyte and CD4^+^ T cell activation, respectively [8]. Compared to lung adenocarcinoma and squamous cell carcinoma, SCLC presents a 5.4 and 6‐fold lower CD8 T cell expression, respectively [22]. In addition, the SCLC microenvironment demonstrates a higher presence of tumour‐infiltrating lymphocytes (TIL) in their stroma rather than the parenchyma, thereby limiting TIL contact with tumour cells [23]. The poor immunogenicity of SCLCs likely prevents a robust response to anti‐PD‐L1 therapy [8]. SCLCs also facilitate a pro‐tumour microenvironment through metabolic mechanisms. SCLCs rely heavily on aerobic glycolysis for ATP production [8] and exist in a highly hypoxic state, [24] creating an acidic and nutrient‐deplete microenvironment poorly conducive to the activities of effector immune cells. In the hypoxic state, SCLC expresses hypoxia‐inducing factor (HIF‐1α), which regulates VEGF‐A‐mediated angiogenesis. HIF‐1a also upregulates immune checkpoint ligands resulting in poorer CD8+ and CD4+ infiltration [25].

## 3 Mutational Patterns

SCLC carries a high somatic mutational burden, predominated by G to T transversions that are highly associated with tobacco‐related carcinogens [5, 26]. Furthermore, SCLC almost universally demonstrates loss of function mutations in the Rb1 and TP53 tumour suppressor genes [5].

Other common genetic patterns observed in SCLC include NOTCH1 mutations (25%), with most mutations being missense (82%), noting that NOTCH normally holds a tumour‐suppressing role in SCLC. Amplifications of the Myc proto‐oncogene family (20%), activating mutations of the oncogenic PIK3CA (15%) and inactivating mutations in the PTEN tumour suppressor gene (9%) also feature with prominence in SCLC [27, 28]. These mutations collectively contribute to cancer hallmarks including that of sustaining proliferative signalling, avoiding immune destruction, enabling replicative immortality, angiogenesis and induction of invasion and metastasis [5, 27].

## 4 The Metastatic Proclivity of SCLC

Notably, SCLC harbours a strong propensity for metastasis activity, [29] commonly to the liver, bones, brain and lymph nodes [30]. Metastasis accounts for 90% of all cancer‐related deaths, [31] and in SCLC, 70% of patients exhibit metastatic disease at the time of diagnosis, [29] with metastatic propensity contributing strongly to poor survivability in SCLC. It is therefore important to understand the biology of metastasis that underpins SCLC to identify potential therapeutic targets against SCLC metastasis. The following sections will address important factors noted to facilitate the metastatic mechanisms of SCLC, also summarised in Table 1.

**TABLE 1:** Summary of the mechanisms of metastasis in SCLC.

Mechanisms of SCLC metastasis				
TIME	Vasculogenesis	EMT	Migration	Organ tropism
Reduced expression of NKG2D ligands: reduced NK cell infiltration	VEGF‐A‐mediated angiogenesis	Transcriptional repression of E‐cadherin	NFIB‐mediated migration	PLGF‐mediated brain metastasis
	VEGF‐C‐mediated lymphangiogenesis	Suppression of Notch signalling, expression of DLL3 (inhibitory Notch ligand)	Expression of selectin ligands CEA, PSGL‐1, CD44	CXCR4‐mediated bone metastasis
	VE‐cadherin‐mediated vascular mimicry	Tumour heterogeneity	E‐/P‐selectin‐mediated migration	
			B1 integrin‐mediated migration	

## 5 Drivers of SCLC Metastasis

### 5.1 Tumour Immune Microenvironment

#### 5.1.1 Poor Natural Killer Cell Infiltration Into the Tumour Immune Microenvironment Leads to SCLC Metastasis

Through murine models, Best et al. demonstrated the pivotal role of natural killer (NK) cells in the control of SCLC metastasis. This is in contrast to CD8+ T cells, where CD8+ T cell depletion did not affect the extent of metastatic dissemination. Best et al. identified a significant reduction of NK cell infiltration in SCLCs relative to peripheral blood mononuclear cells (PBMCs) [32]. Zhu et al. analysed the expression of ligands for the receptor NKG2D, which serves as the most potent activating receptor of NK cells, noting significantly reduced NKG2D ligand expression levels in SCLCs, particularly SCLC‐A subtypes, compared to NSCLCs [33]. Notably, the NKG2D receptor is also expressed in T cells, and the receptor interaction with NKG2D ligands can activate both NK and T cells, which can, respectively, reduce SCLC metastasis and tumour growth, as demonstrated by Zhu et al. through the expression of the NKG2DL ligand in immunocompetent mice models. Analysis of NSCLC and SCLC cell lines by Zhu et al. further revealed a pattern of epigenetic silencing of MICA/B, an important NKG2D ligand, through hypoacetylation, particularly in ASCL1 high/SCLC‐A cell lines. This was reversible in human SCLC cell lines through the use of histone deacetylase (HDAC) inhibitors [33]. There may be benefit in further translational research with HDAC inhibitor therapy, particularly in patients with SCLC‐A, to restore NKG2D ligand expression to enable improved NK and T cell activation.

### 5.2 The Vasculogenic Properties of SCLC

Angiogenesis and lymphangiogenesis feature in SCLC, enabling metastasis through the creation of haematogenous and lymphatic routes of dissemination [34, 35].

#### 5.2.1 VEGF Expression

Human SCLC cell lines express VEGF‐A and VEGF‐C, in addition to their respective receptors VEGFR‐2 and VEGFR‐3 [36] to promote vasculogenesis. The VEGF subclasses can act on their receptors, also expressed by the tumours, in an autocrine fashion [36, 37]. VEGF‐A binds VEGFR‐2 to induce angiogenesis and vascular permeability, [38, 39] and VEGF‐C acts on VEGFR‐3 to facilitate lymphangiogenesis [39, 40]. Canonical tumorigenic pathways such as MAPK or PI3K are often involved in mediating the effector functions of the VEGF family [41]. VEGF‐A enables angiogenesis to create routes for tumour cell dissemination and assist circulating tumour cell survival, extravasation, deposition and colonisation into secondary sites [42]. The lymphangiogenic capacity of VEGF‐C enables lymphatic metastasis, [42] and the expression of VEGF‐C in SCLC cell lines [36] may explain the frequently noted pattern of lymphatic metastasis in SCLC. Exogenous VEGF has been shown to incite SCLC cell proliferation and migration in vitro, [36] reinforcing the understanding of VEGF as a facilitator of SCLC metastasis. Unfortunately, VEGF inhibitors and angiogenesis inhibitors have conferred variable therapeutic effect in SCLC and are not yet mainstays of SCLC treatment [43]. A meta‐analysis of seven randomised controlled trials with a total of 1322 patients did not find an association between angiogenesis inhibitors and improvements in progression‐free survival (PFS), OS or overall response rate (ORR). Subgroup analysis, however, identified that the anti‐VEGF‐A humanised monoclonal antibody Bevacizumab improved PFS (Hazard ratio (HR):0.73, 95% CI: 0.42–0.97, *p* = 0.04) [44]. A later published randomised controlled trial comparing cisplatin plus etoposide with or without Bevacizumab in extensive‐stage SCLC similarly noted the addition of Bevacizumab to increased PFS without increasing OS, although a significant improvement in OS was observed in patients continued on lone Bevacizumab maintenance therapy, provided that they demonstrated an objective response or disease stability following the first six Bevacizumab plus chemotherapy cycles (HR: 0.60, 95% CI: 0.40–0.91, likelihood ratio test: *p* = 0.011) [45]. Accordingly, it may be worthwhile to continue research on Bevacizumab as a maintenance therapy agent in these subpopulations.

#### 5.2.2 Vascular Mimicry

SCLC also exhibits vascular mimicry potential, with vascular mimicry positively associated with invasion and metastasis [46]. Williamson et al. first uncovered the expression of VE‐cadherin in a subpopulation of SCLC circulating tumour cells, noting VE‐cadherin as an important marker of vascular mimicry. They further demonstrated the formation of vascular mimicry networks by SCLCs on matrigel in a VE‐cadherin‐dependent manner, thereby inferring vascular mimicry capabilities in SCLCs. Vascular mimicry is a means of de novo tumour vascularisation [46] where the tumour cells demonstrate plasticity to adopt an endothelial phenotype [47]. In vascular mimicry, the VE‐cadherin transmembrane protein, which is normally present in adherens junctions between endothelial cells, binds tumour cells as they arrange into vessel‐like structures [48]. Vascularisation by vascular mimicry consequently provides additional routes for invasion and metastasis [47]. Currently, there are no VE‐cadherin‐targeting therapies in SCLC, and more research is needed to identify whether there can be a viable role for VE‐cadherin‐targeted therapy in SCLC treatment.

### 5.3 Epithelial‐Mesenchymal Transition

SCLC can enact EMT, and express various ligands and receptors to facilitate invasion and circulatory migration.

#### 5.3.1 Repression of E‐Cadherin as a Hallmark of EMT

EMT is an important step in metastasis, whereby cancer epithelial cells undergo intercellular junction dissociation, loss of apical‐basal polarity, cytoskeletal remodelling and adopt a mesenchymal trait to exhibit a migratory phenotype [49, 50, 51]. Human SCLC lines demonstrate a higher expression of Snail, Slug and Zeb2 than NSCLC lines [50]. These genes are transcriptional repressors of the epithelial marker E‐cadherin, an epithelial marker with an important role in maintaining epithelial cell–cell adherens junctions [51]. Loss of E‐cadherin expression is a hallmark of EMT [51]. Multiple compounds have been shown to upregulate E‐cadherin expression in digestive tract [52] and genitourinary cancers [53] in preclinical studies, and there may be a benefit in extending the research to explore their effects in SCLC.

#### 5.3.2 Notch Signalling in EMT

Notch signalling is often suppressed in SCLC to assist EMT [54]. In the canonical Notch signalling pathway, the Notch transmembrane receptor interacts with its ligands, Delta and Serrate/Jagged, resulting in a proteolytic cleavage of the Notch receptor, releasing the Notch intracellular domain (NICD), which translocates into the nucleus. In the nucleus, NICD complexes with the DNA‐binding protein CSL and a member of the Mastermind‐like (MAML) family of co‐activators to promote the transcription of downstream Notch target genes [55]. Interestingly, the role of Notch signalling in oncogenesis can vary by tumour type. While Notch plays an oncogenic role in most human carcinomas, it appears to be anti‐proliferative in SCLC [54, 56]. Hassan et al. analysed SCLC cell lines with knockdown (KD) of the Notch 1 gene (via Notch 1 small interfering RNA) versus NICD overexpression (via NICD plasmid transfection), noting intercellular dissociation amongst cells with KD Notch1 and increased invasion in their in vitro matrigel invasion assay. A combination of western blotting (WB), immunofluorescence analysis (IFA) and reverse transcription‐polymerase chain reaction (RT‐PCR) showed decreased expression of E‐cadherin, and increased expression of the EMT markers Snail, Slug and Vimentin in cells with KD Notch1. The opposite was true for cells transfected with the NICD plasmid [54]. Altogether, the gene expression and mesenchymal phenotype of KD Notch1 strongly implicates Notch inactivation in SCLC EMT.

#### 5.3.3 Delta‐Like Ligand 3, an Inhibitory Ligand of Notch, Is a Key Orchestrator of SCLC EMT Through the Expression of Snail

Recently, SCLC has been shown to undergo migration and invasion by Delta‐like ligand 3 **(**DLL3)‐mediated upregulation of Snail proteins. DLL3 is an inhibitory ligand of the Notch signalling pathway, and is expressed in over 80% of SCLC surfaces, while being minimally expressed in healthy lung tissues [57, 58]. Nuclear expression of Snail occurs in 31% of SCLC, which represents the highest of all lung cancer subtypes [59]. Furuta et al. demonstrated DLL3 to be an in vitro inducer of Snail expression in SCLC [60]. Snail is widely implicated in EMT by suppressing E‐cadherin, a mediator of cell–cell adhesion [51]. Snail also upregulates mesenchymal markers such as MMP‐9 and fibronectin and downregulates epithelial markers in claudins and occludins [61]. Through migration and invasion assays, Furuta et al. demonstrated significant reduction of SCLC migration and invasion upon Snail downregulation, while DLL3 overexpression increased migration [60]. Snail as an inducer of EMT likely confers invasive properties to SCLC, however It has been shown in breast cancer that Snail can facilitate migration in an EMT‐independent manner, [62] and the potential role for Snail in EMT‐independent migration in SCLC [60] requires further study. The overexpression of DLL3 in SCLC coupled with its minimal expression in normal cells makes DLL3 an ideal therapeutic target. Accordingly, there are several emerging DLL3‐targeted therapies in SCLC [63]. Examples of such therapies include the T‐cell engager therapy Tarlatamab, and the CAR‐T cell therapy AMG 119, which have both shown promising results in preclinical and early clinical studies [63]. The DLL3‐targeted antibody‐drug conjugate rovalpituzumab tesirine (Rova‐T) demonstrated promising phase 1 trial results in SCLC patients with disease relapse after one or two chemotherapy regimens [64]. The phase II trial of Rova‐T however, showed limited efficacy with toxicity concerns [65]. and consequently, its continued development ceased [66].

#### 5.3.4 Tumour Heterogeneity in EMT

The biology of EMT in SCLC is poorly understood, [30, 67, 68] however it is possible that tumour heterogeneity also contributes to its mechanisms of EMT [67]. Krohn et al. observed differing phenotypes of the ‘floating’ cell or ‘adhering’ cell within a single SCLC cell line. The ‘floating’ cells (NCI‐H69) exist as tightly‐packed floating aggregates, exhibiting an epithelial phenotype with high expression of the epithelial markers E‐cadherin and zonula occludens, and an absence of Vimentin—an EMT biomarker and an intermediate filament providing architectural support to cancer cells during migration [67, 69]. Conversely, the ‘adhering’ cells (NCI‐H69V) are characterised by an EMT phenotype, greatly expressing EMT markers such as Vimentin, ZEB1, Snail and Matrix metalloproteinase (MMP)‐2 and 9, with little to no expression of epithelial markers such as zonula occludens and E‐cadherin. The metastatic behaviour of SCLCs may be partially attributed to tumour heterogeneity, whereby the more malignant cells within a tumour drive metastatic behaviour including that of EMT. Krohn et al. further demonstrated a likely role of epigenetic regulation in the differential gene expression, noting significantly lower DNA methylation of the Vimentin promoter and higher methylation of E‐cadherin in NCI‐H69V. Further preclinical studies are needed to assess the viability of these genes as epigenetic therapy targets.

### 5.4 SCLC Cell Migration

#### 5.4.1 The Role of Nuclear Factor I/B in SCLC Migration

The transcription factor Nuclear factor I/B (NFIB) was first recognised by Dooley et al. to possess oncogenic properties in SCLC, [70] and it has since been acknowledged as a driver of SCLC metastasis [71, 72]. In murine models, NFIB is a physiological regulator of lung maturation and brain development [73]. NFIB is highly expressed in human SCLC, [70] especially in metastatic disease, and is correlated with shorter progression‐free survival [71]. In human SCLC cell lines, NFIB binds to DNA and induces chromosome instability by creating a hyper‐accessible chromatin state, permitting increased expression of specific distal gene segments [71]. Interestingly, many genes upregulated by NFIB overexpression and NFIB‐induced transcriptional accessibility are implicated in axon guidance and synaptic arrangement, in addition to cell–cell adhesion and motility [71, 74, 75]. While the neuronal gene expression pattern by NFIB is consistent with its physiological role in brain development, the expression of genes pertaining to developmental neuronal migration are likely also utilised to assist SCLC cells in their migration, a phenomenon that is also seen in the metastatic variants of other cancers [71]. Furthermore, NFIB downregulates E‐cadherin, [72] further implicating its role in invasion and migration [76]. Unfortunately, NFIB is classically an ‘undruggable’ transcription factor [77]. Gao et al. recently identified NFIB as a substrate of the protein arginine methyltransferase CARM1, whereby CARM1 methylates NFIB to promote NFIB transcriptional activity [78]. CARM1 is a clinically viable target, with some small molecule inhibitors of CARM1 having already been identified [79, 80] and CARM1 inhibitor therapy may warrant further research as a means of NFIB repression in SCLC.

#### 5.4.2 Selectin Driven Migration

SCLC expresses ligands for selectin, a cell adhesion molecule, and SCLC also contains selectin binding sites to interact with the endothelium, by which they undergo selectin‐mediated migratory patterns similar to leukocyte migration during inflammation [81, 82]. These selectin ligands include CEA, PSGL‐1 and CD44, while the binding sites include sites for E‐selectin and P‐selectin [81]. Selectins including E‐ and P‐selectin are key regulators of leukocyte adhesion and diapedesis [83, 84]. In particular, SCLC cells adhere strongly to E‐selectin, and were recorded by Richter et al. to roll along E‐selectin coated microslides resembling the rolling pattern of leukocytes along endothelial linings expressing E‐selectin [82]. Such was best represented by the OH1 human SCLC cell line [82]. As later noted by Heidemann et al., in the OH1 cell line‐derived xenograft (CDX) model of SCLC, metastatic capacity is greatly reduced with knockout of E‐/P‐selectin, [81] and it is postulated that selectins can mediate SCLC metastasis by facilitating circulatory transport along the endothelium, mimicking leukocyte rolling patterns. While Heidemann et al. did observe reduced metastasis with E‐/P‐selectin knockdown, the metastatic activity was not completely eliminated, suggesting the likely role of other molecules in the leukocyte adhesion cascade in SCLC migration. Adhesion molecules such as CD24 and Lamp1/2 are still under‐researched regarding their potential role in SCLC metastasis.

#### 5.4.3 B1 Integrin‐Mediated Cancer Cell Migration

Integrins are another set of cell adhesion molecules implicated in solid tumour metastasis, [85] including SCLC [86]. Integrins are comprised of paired A and B subunits, which heterodimerise to form receptors for extracellular matrix (ECM) ligands, [86, 87, 88] and result in the assembly of the adhesome, a molecular network of scaffolding and signalling proteins [87, 89]. In SCLC, the B1 integrin is highly expressed [90] and acts as a pro‐metastatic oncoprotein [86]. The B1 integrin functions to recruit and activate kinases including the FAK and SRC kinases, [87] which are strongly complexed adhesome components [91]. SRC activates FAK, [92] causing disassembly of focal adhesions between the ECM and the cancer cells, triggering cell migration pathways [93]. The role of the B1 integrin in SCLC metastasis was confirmed in studies of human SCLC samples by Zhao et al., extensive‐stage disease was correlated with high B1 integrin expression and low expression of the CUL5 and SCOCS3 proteins, whereby CUL5 and SOCS3 are components of the E3 ubiquitin ligase that normally targets integrin B1 for degradation. Zhao et al. further demonstrated that Dasatinib, an SRC kinase inhibitor used in leukaemia treatment, is associated with decreased proliferation and growth, and increased apoptosis in CUL5‐deficient SCLC CDX tumours [86]. Kinases are tractable targets for therapy [94] and accordingly, Dasatinib may be considered as a clinical trial candidate in CUL5‐deficient SCLC.

### 5.5 Homing of Metastatic SCLC

Secondary site colonisation serves as the final step of the metastatic cascade, [95] and while little is known about the mechanisms by which SCLC metastases preferentially home to their secondary organs, the molecules produced by SCLC can provide explanatory cues about the seeding patterns [30].

#### 5.5.1 PLGF as a Mediator of Brain Metastases

The brain is a common site of SCLC metastasis, with up to 80% of patients experiencing brain metastasis through the course of the disease despite treatment [96]. SCLC brain metastases highly express PLGF. PLGF secreted by SCLC facilitates activation of the Rho kinase, which activates ERK 1/2, leading to disassembly of tight junctions in the blood brain barrier (BBB), enabling seamless transendothelial migration of metastases into the brain. The expression of PLGF may explain the proclivity for SCLC to metastasise into the brain [97].

#### 5.5.2 CXCR4 as a Mediator of Bone Metastases

The bone is another common site for SCLC metastasis, and the expression of the CXCR4 chemokine receptor by SCLC has been correlated with bone‐specific metastasis in preclinical studies.

CXCR4 is highly expressed by SCLC, amongst other cancers, and binds to the CXCL12 ligand [98], which is secreted by osteoblasts of the bone endosteum [99]. The CXCL12‐CXCR4 axis has been demonstrated to facilitate SCLC migration in vitro, [100] and Ma et al. demonstrated that CXCR4 knockout significantly reduced bone metastasis of an injected human SCLC cell line in murine models [100]. As such, the CXCL12‐CXC4 axis potentially mediates a chemotactic migratory pattern of SCLC metastases towards the CXCL12‐secreting bone, however, this postulation pertaining to bone‐specific metastasis still requires further in vivo study.

These examples ascribe to the ‘seed and soil’ theory, where organ‐specific metastasis is strongly influenced by the compatibility between features of the cancer (seed) and the secondary organ (soil) [101].

### 5.6 New Understandings of miRNA as a Potential Therapeutic Target in SCLC Metastasis

While loss of function of P53 and RB are common to most SCLCs, [5] these major tumour suppressors are not classically amenable to therapeutic intervention, and there remains a constant need to identify more therapeutic targets [102]. microRNA (miRNA) targeting has recently been suggested as a possible therapeutic avenue in SCLC [103]. miRNA are short noncoding RNAs bearing translational control over mRNA, and they may be oncogenic or tumour‐suppressing depending on the genes they downregulate [104]. Specific miRNA involved in SCLC proliferation and metastasis is poorly understood. However, Khan et al. have recently identified the miR‐1 miRNA as a tumour suppressor in SCLC, acting on the CXCR4/FOXM1/RRM2 axis to reduce proliferation and metastasis [103].

Through RNA sequencing and biosensing, Khan et al. identified significantly lower miR‐1 in the tumour and serum samples of SCLC patients relative to healthy controls. In their studies, the authors used two main human SCLC lines: SBC3 (an miR‐1 expressing cell line) and SBC5 (a cell line devoid of miR‐1). Variations of the cell lines were also created whereby SBC3 was infected with lentivirus containing the miR‐1Zip (an anti‐miR‐1), to induce SBC3‐miR‐1Zip: a miR‐1‐knockout version of SBC3. Furthermore, SBC5 was placed under the control of a Doxycycline inducible system, whereby Doxycycline could induce the expression of miR‐1 to create an miR‐1 overexpression version of SBC5 (SBC5‐DOX‐On‐miR‐1).

In vitro studies of these cell line variations suggested miR‐1 expression to be strongly associated with decreased tumour growth and decreased migratory ability. These findings were translated in vivo, where SBC3‐miR‐1Zip and SBC5 cells injected into CDX mice models demonstrated significantly greater tumorigenesis and metastasis than SBC3 and SBC5‐DOX‐On‐miR‐1, respectively, with metastasis sites commonly mimicking that of human SCLC metastasis patterns into the liver, brain, bone and lungs. Overexpression of miR‐1 on the other hand, appeared to diminish metastatic activity.

**FIGURE 1:**
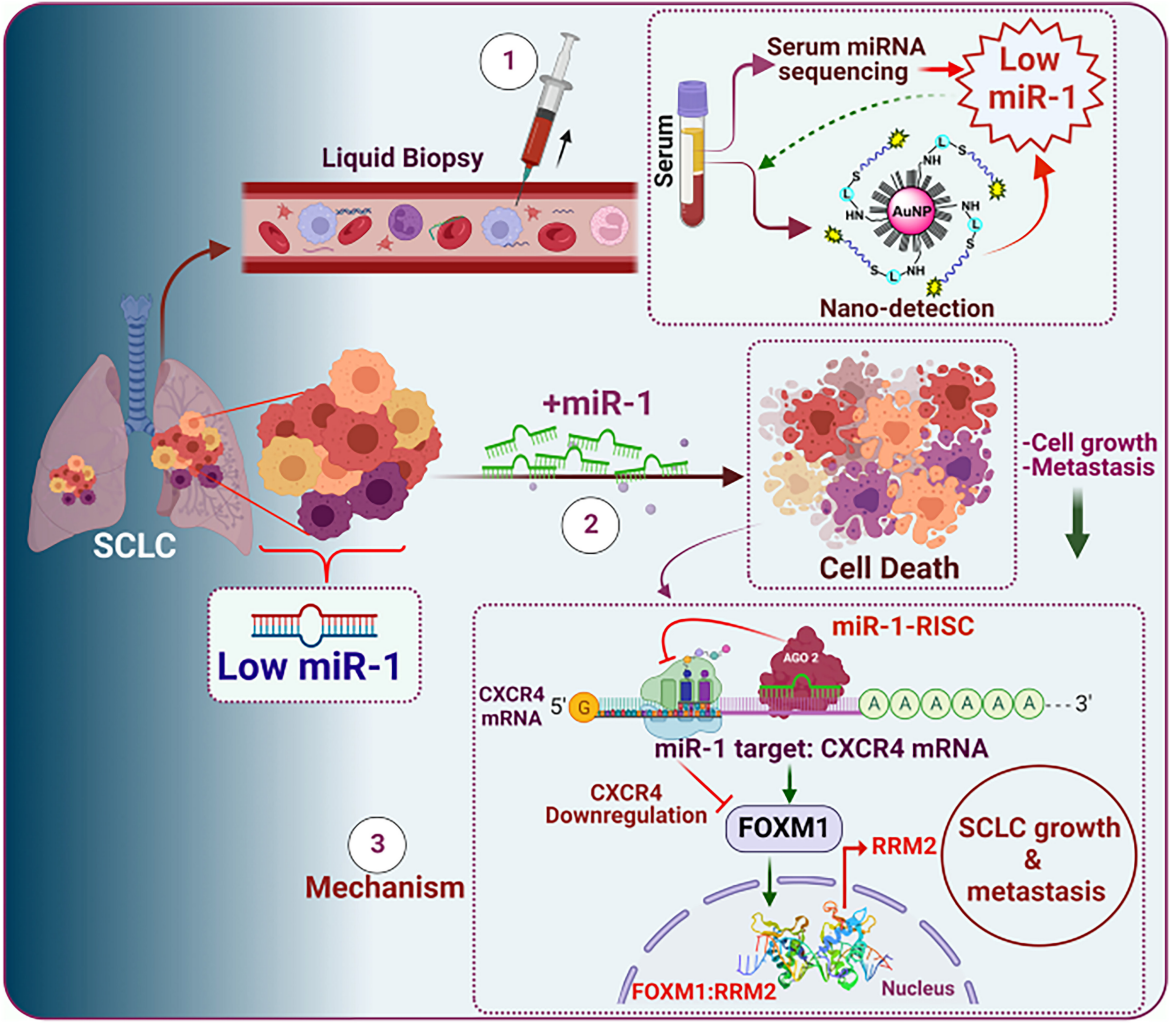
miR‐1 downregulates CXCR4 transcription, resulting in decreased FOXM1‐dependent RRM2 expression, thus limiting SCLC metastatic activity. *Source:* Khan et al. [103]. Creative commons license, source material: https://creativecommons.org/licenses/by/4.0/.

Subsequent RNA sequencing of each cell line variation noted CXCR4 to be the most upregulated gene in cells with low miR‐1. RNA sequencing and a chromatin immunoprecipitation assay also identified the CXCR4, FOXM1 and RRM2 genes to be strongly co‐expressed with downregulation of miR‐1. As previously noted, CXCR4 is commonly upregulated in SCLC, and it is often involved in the metastatic behaviour of various cancers [105]. FOXM1 is a known transcription factor of RRM2, and RRM2 is often implicated in cancer aggression [106]. Through cell surface expression analysis, target scanning and chromatin immunoprecipitation assays, Khan et al. deduced that miR‐1 inhibits CXCR4 expression, which subsequently decreases FOXM1‐mediated RRM2 expression in SCLC. miR‐1 was shown to inhibit expression of metastatic markers such as AKT, ERK and Snail. Expression analyses showed CXCR4, FOXM1 and RRM2 to be highly expressed in liver metastases resected from SCLC murine xenografts. Altogether, they reasoned that miR‐1 expression confers an inhibitory effect on the CXCR4/FOXM1/RRM2 axis, where the axis is otherwise heavily implicated in SCLC metastasis (Figure 1).

Due to its high plasticity and mutational burden, it has been difficult to establish therapeutic targets for SCLC [107]. CXCR4 antagonists are under study as promising means of attenuating SCLC metastasis, [100] and Khan et al. have provided a new understanding of the miRNA miR‐1 as a direct inhibitor of CXCR4 expression, with downstream inhibition of FOXM1/RRM2, leading to reduced SCLC metastasis. As such, there appears to be potential in creating therapies with activating or inducing effects on miR‐1 to limit the metastatic capabilities in SCLC by targeting CXCR4 at the gene expression level.

It should be noted, however, that a single miRNA can regulate several mRNAs, [104] and it is unknown whether therapeutic overexpression of miRNA can result in unintended effects. miR‐1 has a known role in cardiomyocyte development, [108] and its overexpression has been shown to induce cardiac arrhythmias in murine models [109]. Therefore, cardiac safety profiling of potential miR‐1‐inducing therapy for SCLC would be required should miR‐1‐activating drugs be explored in clinical trials.

## 6 Open Questions

Therapeutic research in SCLC is commonly performed on PDX and CDX models, where immunodeficient mice are engrafted with patient‐derived and cell line‐derived tumour tissue, respectively [110]. These immunodeficient mice models, however, do not replicate the TIME [111]. Preclinical studies have identified tumour‐infiltrating macrophages as proponents of SCLC liver metastasis [112] and NK cells as inhibitors of SCLC liver metastasis [32]. The immunodeficient PDX and CDX models do not capture the complex interplay between the immune system and the metastatic activities of SCLC, and there is a great need to develop models that can overcome this experimental challenge. One possible approach may be to reconstitute the human immune system within the mouse model. The Hu‐PBL model involves the engrafting of human leukocytes derived from PBMCs, spleen or lymph nodes into immunodeficient mice, however, the primary limitation is the brief timeframe of 4–6 weeks before the occurrence of lethal xenograft versus host disease, which may not enable adequate time to monitor drug resistance and metastasis activities [113]. An alternative to the immune humanisation of mice models is the injection of human CD34+ haematopoietic stem cells (HSCs) into immunodeficient mice. However, this method also has caveats: not all human haematopoietic lineages develop with full function in these mice models. For example, human B cell differentiation is blocked at the transition phase, and there are functional impairments of the T and NK cells in these models [113]. Patient‐derived organoids (PDOs) have also gained traction in cancer research over recent years, [114] however have not yet become commonly utilised in SCLC research [110]. PDOs are in vitro 3D structures cultivated from enriched cancer cells to closely replicate the micro‐architecture, function and genetic diversity of the original cancer. Advantages of PDOs over PDXs and CDXs include a more accurate replica of the human tumour microenvironment in the assessment of tumour progression and relapse, higher throughput for drug screening, and no concerns of graft versus host disease [110, 114]. Genetically engineered mouse models (GEMMs) have also been used in SCLC [115]. GEMMs are useful in analysing the role of specific driver mutations in the onset and progression of cancer, [115, 116] however do not incorporate the point mutations caused by tobacco‐related carcinogens in SCLC models [115].

As previously noted, the understanding of EMT in SCLC is still lacking. EMT is an evolutionarily conversed step in developmental biology [117] and is often pivotal to tumour invasiveness and consequent metastasis [118]. While Notch signalling is a mediator of EMT in various cancers including NSCLC, [56] Notch appears to exert anti‐EMT properties in SCLC [50]. It is therefore unclear whether any potential therapy that enhances Notch expression might increase the risk of other cancers. Furthermore, Notch signalling has been associated with chemoresistance in SCLC, raising concerns about its viability as a therapeutic target. While DLL3, the inhibitory Notch ligand, has been under investigation for SCLC, there are questions regarding its clinical utility. As noted by Stewart et al., the expression of DLL3 within SCLC tumours is variable and dynamic, which may lead to adaptive resistance to DLL3‐targeted therapy [119]. As such, a deeper understanding of SCLC‐specific Notch and DLL3 signalling is required, should they be pursued as therapeutic targets.

## 7 Conclusion

The hallmark of Invasion and metastasis features greatly in SCLC. SCLC can drive metastasis aided by its TIME, vasculogenic properties, EMT capabilities and expression of migration‐related factors. Furthermore, metastatic SCLC can migrate with organ‐specific tropism. The prognosis of SCLC has seen little improvement over recent decades due to its recalcitrant and metastatic nature, presenting a great challenge to the identification of new therapeutic targets. There are still many gaps in the field of SCLC research. The recent subclassification of SCLC into the SCLC‐A, N, P and Y subtypes according to their differential gene expression highlights the need for more subtype‐specific research into tumorigenesis mechanisms and whether subtype‐specific therapies may provide better outcomes compared to standard generalised therapy. While preclinical trials have identified various biomarkers along the metastatic cascade as contributing to the metastatic properties of SCLC, very few targeted therapies against these markers have entered clinical trials. To reduce the metastatic properties of SCLC, further research may be focused on the viability of possible therapeutic targets including the VEGF family, VE‐cadherin, E‐cadherin, Notch, DLL3, CARM1, E/P‐selectin and B1 integrin. Additionally, emerging areas of research involving epigenetic therapy and miRNA targeting in SCLC may offer more therapeutic opportunities.

## Author Contributions

**Carl He:** conceptualization, investigation, writing – original draft, writing – review and editing, data curation, methodology, visualization.

## Ethics Statement

The author has nothing to report.

## Conflicts of Interest

The author declares no conflicts of interest.

## Data Availability

The author has nothing to report.
